# A Study on the Influence of Traditional Architectural Elements on the Urban Context from the Perspective of Perception: Taking the Yangtze River Delta, the Pearl River Delta, and the Bohai Rim as Examples

**DOI:** 10.1155/2022/1744411

**Published:** 2022-04-20

**Authors:** Tingting Niu, Ruibin Li

**Affiliations:** ^1^School of Architecture and Urban Planning, Anhui Jianzhu University, Hefei 230601, Anhui, China; ^2^School of Architecture, Xi ‘an University of Architecture and Technology, Xi'an 710000, Shaanxi, China

## Abstract

As one of the important components of a city, traditional buildings in different regions just reflect the differences of cities in different regions, and the analysis of the value hierarchy of its constituent elements can reveal the corresponding urban characteristics. This paper selects the representative traditional buildings in the Yangtze River Delta, the Pearl River Delta, and the Bohai Rim region as the research object, takes “perception” as the core concept, combines questionnaires, status surveys, and other methods to obtain and filter traditional architectural elements, and uses the principal components with the help of SPSS software. The analysis method calculates the filtered traditional building elements in the three regions and obtains the principal component composition and hierarchical order of them. Finally, the data results are combined with qualitative analysis to judge the different orders of the traditional building elements in the three regions and their impact on the development of their respective urban contexts.

## 1. Introduction

Perception is the process of gaining awareness or understanding perceptual information [1]. This information originates from the one-way perception of the human body's various sensory cells when they receive environmental features. Gibson summarized perception into five systems: Visual perception system, auditory perception system, olfactory perception system, basic orientation system, and tactile perception system [2]. The premise of a comprehensive understanding of the environment is that the brain combines previous memories and interprets the information obtained by the perceptual system. The formation of cities and buildings is based on spatial perception, which is a real, direct, diverse, and comprehensive perception experience of people to the environment [3].

Spatial perception is affected by the viewing angle (bird's-eye view, parallel viewing angle, head-up viewing angle, etc.) and behavior (walking, driving, and even portable mobile devices) of people's perception of things. Environmental psychology divides the process of human perception of space into four steps, namely “feeling”, “perception”, “cognition,” and “behavior” [4]. Feeling: The process by which human sensory organs receive stimuli from the environment; Perception: On the basis of feeling, with the help of existing knowledge and experience, preliminary comparison and identification of sensory stimuli with the existing cognitive schema in the brain are carried out, thereby forming a comprehensive reflection of things; Cognition: It is the result of emotional processing and logical reasoning on the basis of perception, combined with the perceiver's cultural background, scene situation, thinking ability, etc.; Behavior: Individuals may store perceived environmental information and may also respond with actions, depending on factors such as personal interests, goals, needs, values, and social norms [5].

**Figure 1:**
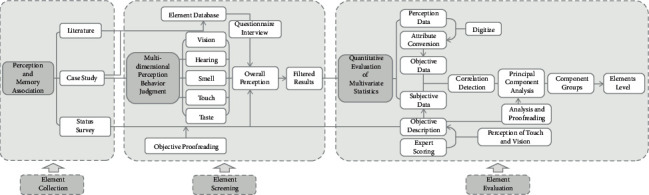
Mind map of perceptual evaluation research on building elements.

From the perspective of perception approach, the spatial perception approach is divided into “experiential” and “constructive.” The former refers to the process of urban experience obtained directly through the bodily senses. The latter is the process of obtaining urban space experience through the reading, processing, and integration of indirect information. In the process of actual environmental cognition, the two usually intersect and jointly promote the subject's perception process of object urban space [6] (Figure 1). “Experiential” perception can serve as a prerequisite for memory in the brain. “Constructive” perception is the main source of imagination provided by the brain, and visual perception is the most important and direct way in which people experience the environment in several perceptual dimensions. Taking visual perception as the starting point and perceiving the image as the carrier, it is a new attempt to analyze the city and architecture by interpreting the information (geographical information and content information) in the image [7]. But in practice, we rarely use one of the senses alone. Instead, they often work together [8]. Therefore, in order to obtain high-quality perceptual features, it is necessary to integrate all human senses (vision, hearing, touch, taste, etc.) to obtain perceptual information [9].

In addition, the study on architectural elements needs to be applied in the concept of the urban context, in order to reflect the influence level of architecture on the development of urban context. The research on context has become a hot topic in the discussion of Western architectural theory in the middle of the last century. Its important historical background stems from the recovery of Western society and economy after World War II and the critique of the functional supremacy of modernism. People advocate that the design of architecture should follow the urban style. The historical relevance emphasizes the building's response and coordination to the surrounding environment. In this regard, Charles Moore's theory of “innumerable measures” [10], Postmodernism, neo-rationalism, and critical regionalism all outline the basic theoretical framework from the essence, analysis, and application of the concept of context and form a preliminary guide for the concept and specificity of architectural elements. After the idea was introduced into China, there was a lot of shock in the field of architecture, and there were also many differences around the definition of the word “CONTEXT.” Mr. Zhou Buyi believes that the word should follow the concept of “environment” and has a negative attitude towards its application and practice in the field of architecture [11], Mr. Zhang Qinnan believes that CONTEXT should be applied to the “virtual” environment in the spiritual and cultural fields, bear the conceptual meaning of “cultural context,” and encourage the contextual relationship between individuals and groups in architectural practice [12]. Therefore, this situation has also caused differences in research results and concepts at home and abroad. In 2005, the domestic scholar Miao Yang comprehensively proposed the establishment of a framework based on the composition and inheritance of urban contextual elements and systematically sorted out the basic composition of urban contextual elements, the principles, contents, standards, and inheritance methods of value evaluation [13]. It clearly depicts the research process of various elements in the urban context, which can be used as a basic framework for the study of architectural elements. Faced with today's complex urban environment, people who live in the city are the core of perceiving the content of various urban elements, but different living environments and knowledge structures will also have different degrees of deviation in the results of perception, which objectively increases. Difficulty in extracting and analyzing perceptual elements. Since the 1970s, the multivariate statistical method represented by principal component analysis has been widely used in the field of urban spatial structure, focusing on analyzing the importance of the representative elements of urban interior space to judge their spatial distribution patterns [14]. Subsequently, the method was more applied to analyze and evaluate the visual attraction and vitality elements of urban landscapes, the vitality evaluation of urban streets, and the measurement of urbanization quality, etc. The results of evaluation research on the elements of urban context are relatively scarce, and there is no form of evaluation yet. Absolute method authority. It is true that in addition to the principal component analysis method, there are also methods such as entropy method and linear regression to analyze various urban indicators in different fields, and the resulting biases are also different. These two methods are good at reflecting the degree of each element to form a single linear order, and the results tend to be more statically determined. However, based on the diachronic characteristics of the context, the dynamic conservation in the process of urban development is emphasized. Therefore, the principal component analysis method categorizes different elements into several “component groups” and maintains independent results, which is more in line with the data model preference of this study.

This case emphasizes the attempt to influence the influence of traditional architectural elements on the urban context in terms of research methods. Firstly, building elements are obtained from multiple channels to form a relatively comprehensive element database. Secondly, the elements are screened according to the survey results of the current situation of traditional buildings in the research site and the questionnaire results of the respondents. (The questionnaire sets options from multiple perception dimensions.) In this way, the factor indicators with a strong perception degree can be obtained. Finally, according to the objective quantitative evaluation of multivariate statistics, more important element levels are generated, which together constitute the thinking framework of “element collection-element screening-element evaluation” (Table 1).

## 2. The Current Situation of the Research Object

### 2.1. Yangtze River Delta Region

**Figure 2:**
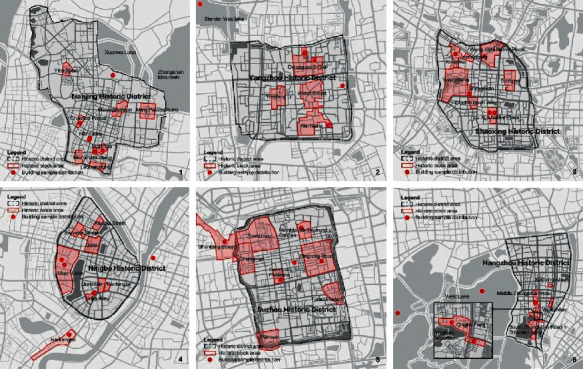
Distribution of traditional historical blocks and building samples in cities in the Yangtze River Delta Region.

The historical and cultural cities of Jiangsu and Zhejiang provinces are the main representatives of the Yangtze River Delta region. The Yangtze River Delta region has densely distributed water networks, crisscrossed river channels, and numerous lakes [15]. Moreover, in the plain area formed by alluvial rivers, the outline of the city conforms to the layout of the landscape, so its shape is relatively free, and the road network in the city shows a state of mixed orthogonal and nonorthogonal. Select Nanjing, Yangzhou, Suzhou in Jiangsu Province and Hangzhou, Ningbo, and Shaoxing in Zhejiang Province as cases. From the perspective of the block level, most of the cases show the characteristics of traditional Chinese style as a whole, which can form a relatively continuous visual image, and the affiliation with the historical urban area is very obvious (Figure 2). Among them, Nanjing, Ningbo, and Hangzhou still have historical blocks with modern western-style due to historical reasons, and the overall preservation quality of the historical blocks is relatively high. However, the atmosphere formed by excessive commercial development has affected the original environmental pattern, and urban development and protective damage have also caused some irreversible consequences [16]. From the perspective of traditional architecture, the architectural heritage of the Ming and Qing Dynasties is the mainstay. Among them, Suzhou, Yangzhou, and Shaoxing have the highest quality preservation and even partially retain the state of the historical human settlements. The individual buildings in Nanjing are well preserved, but in the built-up environment of high-rise buildings, they are slightly isolated and the continuity is not strong. The integrity of part of Hangzhou is relatively unified, but the authenticity of the heritage has been weakened due to the destruction and reconstruction of the protection. The types of buildings are mainly gardens, houses, temples, and other types.

### 2.2. Pearl River Delta Region

**Figure 3:**
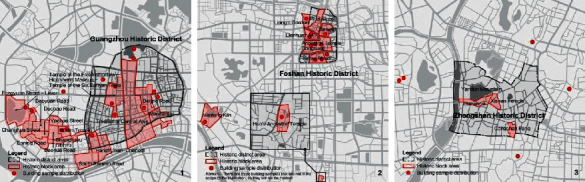
Distribution of traditional historical blocks and building samples in cities in the Pearl River Delta Region.

In the Ming and Qing dynasties, the central Guangdong region with Guangzhou House as the center was the representative region of the traditional Chinese architectural style in the Pearl River Delta region. Among them, Guangzhou, Foshan, Zhongshan, and other historical and cultural ancient cities have the highest quantity and quality of buildings. From the perspective of urban morphology, the city outline of Guangzhou in the Qing Dynasty was close to a semicircle in the north and a rectangle in the south. The road system in the city presents a nonperfectly orthogonal grid [17]. The existing traditional buildings are mostly distributed in the historical urban area formed in the Qing Dynasty (1st in Figure 3). Foshan Chancheng District is the core area of its historical and cultural city. Since the Ming and Qing dynasties, the urban outline has been formed spontaneously based on factors such as natural water systems and human activities, and there are no obvious design traces such as urban axes and road grids [18] (2nd in Figure 3). The urban outline of Zhongshan in the Ming and Qing Dynasties was developed on the basis of Yandun Mountain and Shiqi River, and the modern urban industry and commerce developed rapidly. The expansion and change of the city scale have formed the existing road network and irregular city outline [19], and the distribution of traditional buildings has been separated from the scope of historical urban areas (3rd in Figure 3). From the perspective of the block level, the historical blocks with western style in Guangzhou account for a relatively high proportion, and the Chinese style is distributed near the central axis of the old city. Most of the existing historical blocks in Foshan are dominated by a mixture of Chinese and Western, but the two have destroyed the original texture to a certain extent due to the high-intensity renovation of the old city. The style and appearance of the Zhongshan Historic District is the coexistence of arcades and modern-style buildings during the Republic of China, making the overall style more chaotic. From the architectural point of view, the architectural heritage preserved in the Ming and Qing Dynasties is the main one, among which the architectural style of Guangzhou is well preserved. However, the modern high-rise buildings have formed a squeeze on the ancient buildings, and the living environment in the old city is relatively dilapidated. Foshan's architectural heritage does not have an obvious visual continuous image, and although the surrounding skyline does not form an oppressive situation, the style is slightly mixed. The existing ancient buildings in Zhongshan are scattered and cannot form a continuous style. Most of the architectural types preserved in the three cities are ancestral halls, residences, and temples.

### 2.3. Bohai Rim Region

**Figure 4:**
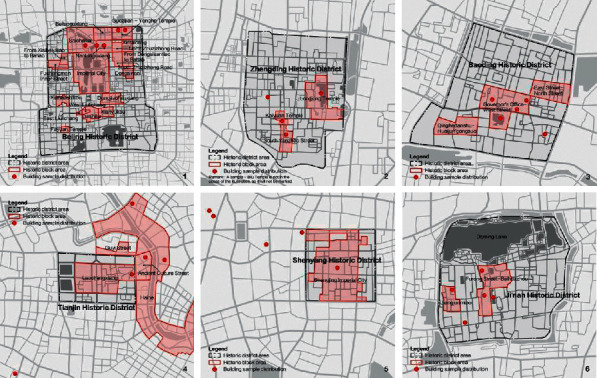
Distribution of traditional historical blocks and building samples in cities in the Bohai Sea Rim Region.

From the perspective of administrative divisions, the Bohai Rim region consists of three provinces and two cities: Beijing, Tianjin, Shandong, Hebei, and Liaoning [20]. Among them, the traditional buildings in Beijing can best reflect the urban characteristics of the northern region. From the perspective of urban morphological characteristics, the outlines of historical urban areas represented by Beijing, Tianjin, Shijiazhuang, Baoding, Jinan, and Shenyang are relatively regular, with a clear urban axis, and the composition is symmetrical, and their road networks are basically orthogonal. From the perspective of the block level, the affiliation with the respective historical urban areas is obvious, showing the traditional Chinese style and features as a whole. Due to the establishment of foreign concessions in modern times, most of the historical districts in Tianjin are western style. Except for Beijing, the status of preservation of historical blocks in other cities shows the characteristics of low proportion and single quantity, and it is difficult to form a block style with strong continuity (Figure 4). Among them, the historical blocks of Jinan and Shenyang are particularly seriously squeezed by the surrounding modern buildings. From the architectural point of view, the proportion of traditional buildings in the Ming and Qing Dynasties is relatively high, and the traditional buildings in Beijing, Baoding, and Zhengding can be traced back to an earlier age. The quality of preservation of traditional buildings in Shenyang is acceptable, but the affiliation with historical blocks is very weak, the distribution is scattered (5th in Figure 4), and the artistic features are not prominent [21]. The scale and grade of the remaining traditional buildings in Jinan are generally not large. The surrounding historical environment is relatively dilapidated and is seriously affected by the commercial atmosphere. The existing high-grade and influential historical buildings in Baoding are of high preservation quality. On the contrary, buildings with lower grades are generally of poorer preservation quality, and the existing number of government buildings is low. Its effect as a city card of “the culture of government office in feudal China” is no longer outstanding. The types of buildings in Shenyang and Zhengding are relatively simple, and the proportion of Buddhist buildings is relatively high. The six cities are generally dominated by temples, dwellings, and mansions.

## 3. Collection and Processing of Traditional Architectural Elements

### 3.1. Collection of Traditional Architectural Elements

Basic data were collected by means of on-site questionnaires. According to the research purpose, “context” and “perception” were used as guiding words when designing the questionnaire. The listed building elements are divided into two levels: explicit and implicit, and the content of elements in each level is divided into two levels, with 9 first-level index items and 30 second-level index items, for a total of 39 index items (Table 2), and set 5 levels according to the importance level for selection (very important, important, general, not important, very not important). Respondents made a grade selection based on their own feelings about the urban environment and their understanding of architectural elements. According to the survey results of the questionnaire, combined with the researchers' perception results of the specific building environment in the field investigation, comprehensive screening was carried out, and the index items with low importance, low environmental perception, and difficult to set quantitative evaluation were eliminated. Finally, a total of 25 index items were extracted. Among them, there are 5 second-level indicators and 20 third-level indicators (Table 3).

### 3.2. Transformation of Traditional Architectural Elements

According to the specific classification of contextual elements in Table 3, all secondary indicators need to be digitally converted to facilitate input into the software for statistical calculation. The basis of digital conversion is the statistical logic of the software and the evaluation degree of the index items, and the numerical order from 0 to 4 is set in turn (Table 4), but the numbers themselves have no numerical meaning, so they will not affect the specific calculation results. All the building samples in Table 1 are in one-to-one correspondence according to the numerical options in Table 4, and finally, the building element index numerical matrix of the building samples in the Yangtze River Delta, the Pearl River Delta, and the Bohai Rim region is formed respectively.

### 3.3. Calculation of Traditional Building Elements

The 15 element indicators listed in the numerical matrix formed by each area are, respectively, input into SPSS19.0 for description and analysis, and the standardized data matrix could be obtained. The total variance contribution rate and the cumulative variance contribution rate of principal components can be obtained by using principal component analysis. This study adopts the method of combining the cumulative variance contribution rate and the eigenvalue to comprehensively determine the number of main factors. The statistical results extracted 6 principal components in each of the three regions and continued to calculate the load structure of the 6 principal components rotated by the maximum variance (in Table 5). The calculation results show that the cumulative contribution rates of the eigenvalues of the 6 principal components in the three regions reach 80.171%, 79.260%, and 75.045%, respectively, which means that the 6 principal components can explain most of the effective information of the 15 original indicators.

Extraction method: principal component analysis: in the Yangtze River Delta region, the first principal component basically reflects the “topographic features, water system, wall color and ventilation” of traditional buildings. The second principal component basically reflects the “roof color, decorative details, architectural style” of traditional buildings. The third principal component basically reflects the “door head and window lintel, material composition” of traditional buildings. The fourth principal component basically reflects the “layout and roof form” of traditional buildings. The fifth principal component basically reflects the “orientation and ethnic customs” of traditional architecture. The sixth principal component basically reflects the “structural form and building age” of traditional buildings.

In the Pearl River Delta region, the first principal component basically reflects the “ventilation/daylighting, material composition, architectural style, and decorative details” of traditional buildings. The second principal component basically reflects the “ethnic customs, roof color and wall color” of traditional architecture. The third principal component basically reflects the “roof form, door head and window lintel” of traditional buildings. The fourth principal component basically reflects the “orientation and layout” oftraditional buildings. The fifth principal component basically reflects the “topographic features and water system” of traditional buildings. The sixth principal component basically reflects the “building age and structural form” of traditional architecture.

In the Bohai Rim region, the first principal component basically reflects the “roof color, ethnic customs, layout, ventilation/lighting” of traditional buildings. The second principal component basically reflects the “wall color, decorative details and architectural style” of traditional buildings. The third principal component basically reflects the “door head and window lintel, material composition” of traditional buildings. The fourth principal component basically reflects the “structural form and roof form” of traditional buildings. The fifth principal component basically reflects the “orientation and building age” of traditional buildings. The sixth principal component basically reflects the “topographic features and water system” of traditional buildings (Table 6).

## 4. Hierarchical Differences of Traditional Architectural Elements

According to Table 7, it can be clearly seen that the distribution and ordering of traditional architectural elements in the three areas are both the same and different at each level. For example, in level 1, the “ventilation/daylighting” factor indicator is reflected in all three areas. Although the specific expressions are different, it reflects that traditional Chinese culture and construction concepts attach more importance to the adaptability of “climate.” In tier 5 and tier 6, the indicator of “building age” occupies almost the same.

Position among the three, which also shows that the influence degree of this factor, is similar in the three regions. Limited by objective factors such as the preservation status of the building, on the one hand, the cases of architecture are mostly concentrated in the Ming and Qing Dynasties, which has a certain unity, which is in line with the “synchronicity” characteristic of cultural heritage. On the other hand, this indicator is relatively low in the overall hierarchical ranking, and its impact on the city is not important, and it also conforms to the “diachronic” characteristic of cultural heritage. In level 5, the “orientation” index is in the same order in the Yangtze River Delta and the Bohai Rim region, and the expressions are both “north-south.” This reflects the universality of traditional construction concepts and traditional customs, so its importance level is relatively low. In the Pearl River Delta, this indicator is relatively high, and its manifestations are also different, reflecting the impact of the local natural environment and social environment on the building orientation. Therefore, its ranking is relatively high.

The differences in the indicators of traditional architectural elements at different levels in the three regions just reflect the different emphases of urban cultural development in each region. From the perspective of a higher level of influence on the development of urban context, the Yangtze River Delta region is most affected by the factors of “topographic features, water system, wall color, ventilation/daylighting.” It is embodied in “mainly flatland,” “ near water or waterfront,” “mainly white,” “patio or small-scale courtyard,” mostly based on local objective natural geographical features and local cultural thoughts. The Yangtze River Delta region is affected by the impact plains and dense water network in the middle and lower reaches of the Yangtze River. The layout of the buildings has a high intimacy with water and the terrain is relatively flat. It is also radiated by regional central cities such as Nanjing. As a gathering place for Jiangnan students, the aesthetics formed by Confucianism and Taoism are clearly reflected in the architecture, and the regional characteristics are extremely high. Secondly, it is affected by factors such as architectural color, decoration, materials, and so on. This reflects that in the context of a relatively developed economy, the long-term development, and integration of the prosperity of local culture and the high degree of exquisite construction technology, it reflects very exquisite decoration and door head.

The Pearl River Delta region is most affected by the elements of “ventilation/daylighting, material composition, architectural style, and decorative details,” which are reflected in “patio, small-scale courtyard, veranda,” “masonry, wood,” “Lingnan regional style,” “ Rich and ornate decoration.” It is mainly affected by the objective factors of climate, economic population size, regional culture, and local craftsmanship. The performance of its building materials and styles is the most prominent than the other two places, and the local kaolin clay walls are mostly used, and the colors are warmer. It is deeply influenced by Shanghai-style culture, and the architectural colors do not reflect strong Confucian aesthetics but also combine local natural imagery or visual coolness to form a blue-green roof color. In terms of decoration, it is more inclined to the carving of mythical figures, with bright colors, so the importance of other elements with “color and customs” as the key words is close behind, which makes it the most characteristic of the three regional urban contexts. Obviously, similar to the Yangtze River Delta region, it attaches great importance to the ventilation effect of the building, and the methods are more eclectic, which shows that the climate characteristics of the two are relatively similar humid and hot environments.

The Bohai Rim region was affected by the environment of Beijing and Shenyang, two capitals during the Ming and Qing Dynasties. The “decoration,” “color,” “scale,” and “culture” of its buildings not only reflect the combination of early Manchu traditional culture and urban context but also reflect the traditional characteristics of the Han nationality. Under the radiation of the north as the center of political imperial power, the number of high-level buildings is constantly increasing, and the hierarchical relationship between buildings is constantly strengthening. “Roof form,” “decorative details,” “roof color,” and “wall color” are all distributed in Level 1 and Level 2. The proportion of gold and red in the buildings is higher, and the scale and level are larger than those of the other two places. Factors such as “religious customs” and “axial layout with due south and north” are ranked higher, which reflects that the strict system, hierarchy, and scale of the northern political center are the dominant factors. Although the “ventilation/daylighting” index is at the same level as the other two places, the Bohai Rim region is affected by the cold and dry climate, and more emphasis is placed on the lighting and heating of the building. Therefore, more attention is paid to the design of the inner courtyard, which is more in line with the architectural style and customs caused by the radiation of the political center in the northern region, which has the highest commonality among all architectural samples in the northern region.

**Figure 5:**
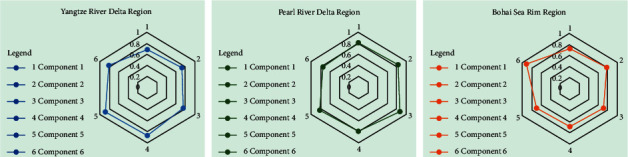
Principal component mean distribution results by regions.

In addition, according to the eigenvalues of each index in Table 6, the eigenvalues of each principal component can be calculated to obtain the mean distribution results of principal components in each region (Figure 5). From the results, the graphs in the Pearl River Delta region are relatively balanced, indicating that the degree of difference between the principal components is relatively average, and the score of the factor indicators in each component is average, so the differences in the degree of development of the urban context reflected in the indicators are also relatively balanced., the linear relationship of urban cultural heritage is relatively relaxed, which can not only highlight the elements with higher importance, but also take care of the elements with lower importance. The graphic balance of the Yangtze River Delta region is second, and the difference between the first three principal components and the last three principal components is large, indicating that the development balance of the high-influence index group and the low-influence index group is poor, and the urban context There is an inflection point in the linear relationship of inheritance, which can moderately increase the influence of the factor indicators in the first three principal components on the inheritance of urban cultural context and raise the inflection point in the development level and improve the balance. The figure balance in the Bohai Rim region is the lowest, and the difference between principal components 1, 2 and principal components 3, 4, 5, and 6 is relatively large, especially the principal component 6 shows the highest degree of difference. The development balance of each group index is poor, reflecting that there are many inflection points in the linear relationship of urban cultural heritage. In particular, it is necessary to focus on amplifying the effect of high-influence index groups in inheritance. Relatively, it cannot be ignored. The effectiveness of low-index groupings will improve the balance of the development of various indicators.

## 5. Conclusion

This paper combines quantitative and qualitative analysis methods to obtain the hierarchical relationship of the elements of urban traditional architecture, reflecting the influence of different elements on the inheritance of urban context. And through the manifestations of each element index at different levels in each city, the differences in cultural characteristics between different regional cities are reflected. The research lists the architectural elements that affect the development of urban context through multichannel methods such as literature reading and field investigation and integrates a relatively comprehensive set of element indicators. Questionnaires are distributed to the people in the study area to obtain the factor indicators with a high degree of perception and statistical integration. Combined with the on-the-spot investigation of traditional building samples in each study area, the current situation information is obtained and sorted. Combined with the index results generated by the questionnaire survey, a number of statistical methods are used to screen and integrate the indicators to form the final element index set. According to the combination of expert evaluation and data analysis, the element indicators in the three regions are integrated and ranked in a quantitative way. And analyze the influence of different levels of elements on the development of urban context so as to show the difference in the focus of urban context development in the Yangtze River Delta, the Pearl River Delta, and the Bohai Rim. Analyzing and researching fragments of urban history as urban context nodes can deepen the interpretation of the city itself and its culture. When carrying out new urban practice in this way, it should be connected with the urban context and appropriately amplify its unique cultural characteristics to enhance the city's recognition. This has certain reference and reference significance to avoid stepping into a predicament, which is that many cities have the same appearance.

## Figures and Tables

**Table 1:** Comparison of information on traditional building samples in Region of Yangtze River Delta, Pearl River Delta, and Bohai Rim.

Regions	Yangtze River Delta Region									
City	Nanjing					Yangzhou				
Architectural samples	Fuzimiao	Zhang yuan garden	Ganxi house	Chaotian palace	Jiming temple	Daming temple	Geyuan garden	Wu's residence	He garden	Wudang xinggong
Historic block	Fuzimiao	Fuzimiao	Nanbuting	Chaotian palace	None	Slender Westlake	Dongguan street	None	Nanhexia	Dongguan street
Category	Temple	Garden	Residence	Palace	Temple	Temple	Garden	Residence	Garden	Temple
Protection level	Municipal level	National level	National level	National level	Municipal level	National level	National level	National level	National level	Provincial-level
City	Shaoxing					Ningbo				
Architect-ural samples	Lu xun ancestral residence	Huajia Taimen	Lu's residence	Cai Yuanpei's former residence	Sanwei Shuwu	Tianyi pavilion	Qing'an Guildhall	Yuan Muzhi's former residence	Yang Fang's former residence	Yuehu Mosque
Historic block	Lu xun Native place	Shimenkan	Xixiaohe	Wang xizhi Native place	Lu xun Native place	Moon lake	None	Nantanghe	Yujia Alley	Moon lake
Category	Residence	Residence	Residence	Residence	Private School	Library	Guildhall	Residence	Residence	Mosque
Protecti-on level	National level	Municipal level	National level	National level	National level	National level	National level	Municipal level	Municipal level	Provincial level
City	Suzhou					Hangzhou				
Architect-ural samples	Taoist Trinity Hall of xuanmiao temple	QuanJin Guildhall	Xu's residence	Humble Administ-rator's garden	Garden ofPleasance	Hu Qing yu Tang	Wen Lan pavilion	Phoenix Mosque	Lingyin temple	Hu Xueyan's former residence
Historic block	None	Pingjiang road	Shantang street	Humble Administrator's garden	Garden ofPleasance	Qinghe Fang	West lake	Middle Zhongsha-n road	West lake	QingheFang
Category	Temple	Guildhall	Residence	Garden	Garden	Store	Library	Mosque	Temple	Garden
Protecti-on level	National level	National level	Municipal level	National level	Provincial level	National level	National level	National level	National level	National level
Regions	Pearl river Delta region									
City	Guangzhou									
Architectural samples	Chen Clan ancestral temple	Guangxia-o temple	Huaisheng Mosque - wang yuelou	Temple of the six BanyanT-rees - Hall of Guanyin	Temple of the five Immortals	Zhenhai Tower	Sanyuan palace	Dafo temple - Mahavira Hall	Haopan street Mosque	Guangzhou city God temple
Historic block	None	None	Temple of the five Immortals - Huaisheng Mosque - temple of the six Banyan Trees			Traditional central axis			Haizhu'n-an - Changdi	Beijing road
Category	Ancestral temple	Temple	Gateway	Temple	Taoist temple	Gateway	Taoist temple	Temple	Mosque	Taoist temple
Protection level	National level	Protection level	National level	Protection level	National level	Protection level	National level	Protection level	National level	Protection level
City	Guangzhou					Zhongshan				
Architectural samples	The Myriad Trees Academy	Hualin temple	Architectural samples	The Myriad Trees Academy	Hualin temple	Yanzhou Academy	Changzhou Huang's ancestral temple	Main Hall of Baiyi temple	Sanshan temple	Dongyue temple
Historic block	Beijing road	Hualin temple		Fengyuan street - Liwan		None				
Category	Academy of Classical Learning	Temple	Guildhall	Taoist temple	Ancestral temple	Academy of Classical Learning	Ancestral temple	Temple	Temple	Temple
Protecti-on level	National level	Municipal level	Provincial level	Provincial level	Municipal level	Provincial level	Provincial level	Municipal level	Municipal level	Municipal level
City	Foshan									
Architect-ural samples	Foshan ancestral temple	Jiuhang Guildhall	Wenhuili Jiaquwu	Liang's garden	Ren wei	Lin's ancestral temple	Huo's ancestral temple	GuogongTemple	Zhaoxiang Huang ancestral temple	Qinghui garden
Historic block	Foshan ancestral temple - Donghuali			Liang's garden	Ren wei	Nanfeng Kiln	Huo's ancestral temple	Xin'an street	None	None
Category	Temple	Store	Residence	Garden	Residence	Residence	Ancestral	Temple	Ancestral temple	Garden
Protecti-on level	National level	Municipal level	Provincial level	Provincial level	Municipal level	Provincial level	Provincial level	Municipal level	Municipal level	National level
Regions	Bohai Sea Rim region									
City	Beijing					Shijiazhuang				
Architect-ural samples	Guozijian	Yonghe temple	Drum Tower	Qi Baishi Memorial Hall	Prince Gong's mansion	Zhengding Longxing temple	Bell Tower of Kaiyuan temple	Pilu temple	Zhengding Confucian temple	Liang's ancestral temple
Historic block	Guozijian - yonghe temple	Guozijian - yonghe temple	Shichahai	Nanluoguxiang	Shichahai	Longxing temple	Kaiyuan temple	None	None	South yanzhao street
Category	Academy	Temple	Gate Tower	Residence	Mansion house	Temple	Temple	Temple	Temple	Ancestral temple
Protecti-on level	National level	National level	National level	Municipal level	National level	National level	National level	National level	National level	Provincial level
City	Baoding					Tianjin				
Architec-tural samples	Daci pavilion	Baoding west Mosque	Bell Tower	Gulianchi	Zhili Governo-r's office	Tianjin Confucian temple	Li Shutong's former residence	Prince Zhuang's mansion	Yuhuang temple	Dabei temple
Historic block	Governo-r's office - west street	None	Governo-r's office - west street	Governo-r's office - west street	Governo-r's office - west street	Laochengxiang	Haihe	None	Ancient culture street	Haihe
Category	Temple	Temple	Tower	Garden	Government office	Temple	Residence	Mansion house	Taoist temple	Temple
Protecti-on level	National level	Municipal level	National level	National level	National level	Municipal level	Municipal level	National level	Municipal level	Municipal level
City	Shenyang					Jinan				
Architec-tural samples	Shenyang imperial palace	Shenyang south Mosque	Xibo ancestral temple	Taiqing temple	Shisheng temple	Luzu temple	Gao's pawnshop	Fuxue Confucian temple	Shandong Governor's office	Guandi temple
Historic block	Shengjing imperial city	None	None	None	None	Historic district	Shengjing imperial city	None	Historic district	Shengjing imperial city
Category	Palace	Temple	Residence	Taoist temple	Temple	Temple	Store	Temple	Governm-ent office	Temple
Protecti-on level	National level	Municipal level	National level	Provincial level	Provincial level	Provincial level	Provincial level	Provincial level	Provincial level	Provincial level

**Table 2:** Basic data of building elements.

Indicator Layer		First-level Indicators	Second-level Indicators
Architectural aspects	Explicit	Site condition, business form, architectural form, architectural color, structural system, passive design	Topographic features, water system, climate, surrounding facilities, environmental atmosphere, architectural style, layout, physical features, roof form, door head and window lintel, wall color, roof color, decorative details, building volume, structural form, material composition, architecture Material, construction method, support system, partition system, orientation, daylighting/ventilation, temperature and humidity
	Implicit	Architectural background, social background, industrial economy	Building age, ethnic customs, religious beliefs, historical events, population composition, economic structure, industrial model

**Table 3:** Final filter data for building elements.

Indicator Layer		First-level Indicators	Second-level Indicators
Architectural aspects	Explicit	Site condition, architectural form, structural system, passive design	Topographic features, water system, architectural style, layout, roof form, door head and window lintel, wall color, roof color, decorative details, structural form, material composition, orientation, daylighting/ventilation
	Implicit	Architectural background	Building age, ethnic customs

**Table 4:** Digital assessment framework for each element indicator.

Nature of Indicators	Primary Indicators	Secondary Indicators	Factor Carrier	Digital Assessment Transformation
Explicit	Site condition	Topographic features	Built-up base situation	Depression-1 Flatland-2 Slope-3
		Water system	Relationship with water systems	No water-0 Distant water-1 near water-2 Adjacent water-3
	Architectural form	Architectural style	Traditional styles of architecture	Regional style-1 traditional Chinese official style-2
		Layout	Plan and general plan outline style	Centralized type-1 distributed type-2Axial type-3
		Roof form	Number of slopes on the roof	Double-slope-1 four-slope-2Double slope and four slopes coexist-3
		Door head and window lintel	Degree of design of the door head and window lintel	None-0 Simple-1 Rich-2
		Wall color	Wall main color	White-1 green brick or warm gray-2Red or yellow-3
		Roof color	Roof main color	Green gray tile-1 color tile-2
		Decorative details	Ridge, cornice architrave, carving, special symbols, etc.	None-0 Simple-1 Rich-2
	Structural system	Structural form	Types of concrete practices for timber construction	Column and tie construction-1Post and lintel construction-2Mix of different wooden structures-3Brick and wood structure-4
		Material composition	Composition of the main material of the building	Wood-based-1 Brick or stone based-2Mixed brick and wood-3
	Passive design	Orientation	Orientation of the main building	North-south direction-1 East-west direction-2
		Daylighting/Ventilation	Layout measures of daylighting and ventilation	Patio-1 inner courtyard-2 Outer corridor-3 cold alley-4

Implicit	Architectural background	Building age	Era of construction of the main building	Qing Dynasty-1 Ming Dynasty-2Before Ming Dynasty-3
		Ethnic customs	Local customs and activities	None-0 Religious-1 Folklore-2

**Table 5:** Rotation sums of squarded loadings contrast by regions.

Component	Yangtze River Delta Region			Pearl River Delta Region			Bohai Sea Rim Region		
	Total	% of Variance	Cumulative %	Total	% of Variance	Cumulative %	Total	% of Variance	Cumulative %
1	2.191	15.651	15.651	3.856	25.710	25.710	2.450	16.330	16.330
2	2.100	14.998	30.649	1.987	13.248	38.957	2.118	14.121	30.451
3	1.801	12.868	43.517	1.857	12.382	51.339	2.006	13.375	43.827
4	1.752	12.512	56.029	1.509	10.063	61.402	1.963	13.088	56.914
5	1.702	12.154	68.183	1.426	9.509	70.911	1.361	9.074	65.988
6	1.678	11.988	80.171	1.252	8.349	79.260	1.359	9.057	75.045

**Table 6:** Contrast of components sequence by regions.

Component	Component 1	Component 2	Component 3	Component 4	Component 5	Component 6
Yangtze river Delta region	Topographic features 0.83	Roof color 0.83	Door head and window lintel 0.87	Layout 0.89	Orientation 0.96	Structural form 0.82
	Water system 0.67	Decorative details 0.71	Material composition 0.62	Roof form 0.84	Ethnic customs 0.80	Building age 0.78
	Wall color 0.65	Architectural style 0.66				
	Daylighting/Ventilation 0.60									
Pearl river Delta region	Daylighting/Ventilation 0.91	Ethnic customs 0.91	Roof form 0.93	Orientation 0.84	Topographic features 0.91	Building age 0.79
	Material composition 0.83	Roof color 0.83	Door head and window lintel 0.82	Layout 0.74	Water system 0.72	Structural form 0.71
	Architectural style 0.80	Wall color 0.79				
	Decorative details 0.74									
Bohai Sea Rim region	Roof color 0.85	Wall color 0.82	Door head and window lintel 0.85	Structural form 0.76	Orientation 0.70	Topographic features 0.91
	Ethnic customs 0.71	Decorative details 0.81	Material composition 0.57	Roof form 0.61	Building age 0.69	Water system 0.90
	Layout 0.69	Architectural style 0.71				
	Daylighting/Ventilation 0.65									

**Table 7:** Comparison of the differences in the stratification of factor indicators by regions.

Level	Region								
	Yangtze River Delta Region			Pearl River Delta Region			Rim Bohai Sea Region		
	Factor Indicators	Expressions	Influencing Factors	Factor Indicators	Expression	Influencing Factors	Factor Indicators	Expression	Influencing Factors
Level 1	Topograph-ic feature	Mainly flatland	Physical geography	Daylighting/Ventilation	Inner courtyard/Patio/exterior porch	Climate	Roof color	Gold/greenish gray	Culture/grade
	Water system	Near water/waterfront	Physical geography	Material Compositi-on	Mixed brick, stone and wood	Local culture	Ethnic customs	Religion/Folklore	Local culture
	Wall color	White	Local culture	Architectu-ral style	Lingnan region	Local culture	Layout	Axial	Regime/Culture
	Daylighting/Ventilation	Interior courtyard/patio	Climate	Decorative details	Rich and beautiful	Local craftsmans-hip/culture	Daylighting/Ventilation	Inner courtyard	Climate
Level 2	Roof color	Gray predomina-ntly	Grade/Culture	Ethnic customs	Religion/Folklore	Local culture	Wall color	Red/brick color	Grade/culture
	Decorative details	Rich and plain	Local craftsmans-hip/culture	Roof color	Lime green/gray	Grade/local culture	Decorative details	Rich and gorgeous	Ethnic culture/craftsmans-hip
	Architectu-ral style	Jiangnan region	Local craftsmans-hip/culture	Wall color	Green brick color/warm gray	Physical Geography	Architectural style	Traditional Chinese official style	Historical background
Level 3	Door head and window lintel	Exquisite modeling	Local craftsmans-hip/culture	Roof form	Four-slope/double-slope	Grade/scale	Door head and window lintel	Less and simple	Regime/craftsmans-hip
	Material Compositi-on	Mixed brick and wood	Local Craftsman-ship	Door head and window lintel	Simple styling	Local culture	Material Compositi-on	Wood-based Constructi-on	Scale/craftsmans-hip
Level 4	Layout	Axial type	Terrain/traditional customs	Orientation	Multi-directional	Natural environme-nt/social environme-nt	Structural form	Column and tie constructi-on mainly	Scale
	Roof form	Four-slope and rich in form	Scale/culture	Layout	Axial type	Traditional Ritual	Roof form	Four-slope	Grade/scale
Level 5	Orientation	North-south	Traditional customs	Topograph-ic feature	Flatland	Physical Geography	Orientation	North-south	Traditional customs/climate
	Ethnic customs	Religion/Folklore	Local culture	Water system	Distant water	Artificial transformation	Building age	Ming and Qing dynasties mainly	Preservati-on status
Level 6	Structure form	Column and tie constructi-on/post and lintel constructi-on	Scale/function	Building age	Qing Dynasty	Preservati-on status	Topograph-ic feature	Flatland	Physical Geography
	Building age	Ming and Qing dynasties	Preservati-on status	Structure form	Column and tie constructi-on/Post and lintel constructi-on	Scale	Water system	Near water/Far water	Physical Geography

## Data Availability

Data sharing is not applicable to this article as no new data were created or analyzed in this study.
